# Diagnostic capability of dynamic ultrasound evaluation of supination-external rotation ankle injuries: a cadaveric study

**DOI:** 10.1186/s12891-019-2899-z

**Published:** 2019-10-30

**Authors:** Cara L. Fisher, Tebyan Rabbani, Katelyn Johnson, Rustin Reeves, Addison Wood

**Affiliations:** 1 University of North Texas Health Science Center, 3500 Camp Bowie Blvd, Fort Worth, TX 76107 USA; 2 John Peter Smith Hospital, 1500 S Main St, Fort Worth, TX 76104 USA

**Keywords:** Syndesmosis, Ultrasonography, AITFL, Ankle fracture

## Abstract

**Background:**

Ankle syndesmosis injuries are common and range in severity from subclinical to grossly unstable. Definitive diagnosis of these injuries can be made with plain film radiographs, but are often missed when severity or image quality is low. Computed tomography (CT) and magnetic resonance imaging (MRI) can provide definitive diagnosis, but are costly and introduce the patient to radiation when CT is used. Ultrasonography may circumvent many of these disadvantages by being inexpensive, efficient, and able to detect injuries without radiation exposure. The purpose of this study was to evaluate the ability of ultrasonography to detect early stage supination-external rotation (SER) ankle syndesmosis injuries with a dynamic external rotational stress test.

**Methods:**

Nine, all male, fresh frozen specimens were secured to an ankle rig and stress tested to 10 Nm of external rotational torque with ultrasonography at the tibiofibular clear space. The ankles were subjected to syndesmosis ligament sectioning and repeat stress measurements of the tibiofibular clear space at peak torque. Stress tests and measurements were repeated three times and averaged and analyzed using a repeated one-way analysis of variance (ANOVA). There were six ankle injury states examined including: Intact State, 75% of AITFL Cut, 100% of AITFL Cut, Fibula FX - Cut 8 cm proximal, 75% PITFL Cut, and 100% PITFL Cut.

**Results:**

Dynamic external rotation stress evaluation using ultrasonography was able to detect a significant difference between the uninjured ankle with a tibiofibular clear space of 4.5 mm and the stage 1 complete injured ankle with a clear space of 6.0 mm (*P* < .02). Additionally, this method was able to detect significant differences between the uninjured ankle and the stage 2–4 injury states.

**Conclusion:**

Dynamic external rotational stress evaluation using ultrasonography was able to detect stage 1 Lauge-Hansen SER injuries with statistical significance and corroborates criteria for diagnosing a syndesmosis injury at ≥6.0 mm of tibiofibular clear space widening.

## Introduction

The ankle syndesmosis, or distal tibiofibular joint, functions to conjoin the tibial and fibular malleoli to form the ankle mortise through four ligaments: anterior inferior tibiofibular ligament (AITFL), posterior inferior tibiofibular ligament (PITFL), interosseous ligament (IOL), and the inferior transverse ligament (ITL). The deltoid ligament, although not one of the ligaments primarily responsible for the stability of the syndesmosis, is often involved in supination-external rotation (SER) injuries. Injuries to the syndesmosis are commonly concomitant in up to 23% of all ankle fractures and involved in up to 10% of all ankle sprains [1]. These ligaments keep the talus well seated between the tibia and fibula by maintaining mortise integrity. When the syndesmosis is injured, it allows greater movement of the talus within the mortise and decreases the contact surface area in the ankle, although this depends on injury severity [2]. This can cause decreased function due to pain and instability and may lead to accelerated degradation of cartilage and formation of osteoarthritis [3].

Ankle syndesmosis injuries are common, but difficult to diagnose and treat. Proper treatment of syndesmosis injuries requires accurate diagnosis to prevent the long-term sequelae of osteoarthritis and decreased function from biomechanical changes and pain. Plain film radiographs and stress fluoroscopy are the traditional diagnostic modalities of choice, but current literature has shown lower sensitivity and specificity than initially perceived [4, 5]. For ankle syndesmosis injuries, appropriate reduction of the fibula in the incisura is essential for proper treatment and requires imaging or direct visualization to verify. Traditional plain film radiographs and fluoroscopy have shown to be inconsistent methods for accurate diagnosis and fibular reduction verification [4, 6]. Most commonly, plain film radiographs and stress fluoroscopy are still used to initially evaluate the ankle syndesmosis with advanced imaging being reserved for subtle cases with high clinical suspicion. However, definitive diagnosis can be made with computed tomography (CT) and magnetic resonance imaging (MRI), which are costly and require the patient to receive radiation with the use of CT. Diagnostic capabilities must be refined to improve cost efficiency and diagnostic accuracy to avoid long term sequelae of syndesmotic diastasis and improve outcomes by identifying malreduction.

Our central hypothesis was that dynamic ultrasonography would be able to consistently and accurately detect early stage SER syndesmosis injuries of stage 1 and 2 (Table 1). An in-vitro simulation using fresh frozen cadaveric material was utilized to test this hypothesis incremental injuries to the ankle syndesmosis for this experiment. The purpose of this study was to validate the ability of dynamic ultrasonography stress examination to detect Lauge-Hansen supination-external rotation (SER) syndesmosis injuries through measurement of tibiofibular clear space measurements.

## Methods

### Specimen preparation

Eleven, all male, fresh frozen specimens were obtained from the UNT Health Science Center Willed Body Program and the UT Southwestern Willed Body Program. These specimens were thawed and mounted into the ankle rig via four steinmann pins placed into the tibia. These pins avoided the fibula and ensured free movement of the fibula at all times. A lateral Kocher approach, done by incising the skin inferior and posterior to the fibula, was used keeping intact all musculature and releasing the inferior flexor retinaculum. Electromagnetic tracking sensors were placed on the tibia and fibula using nylon screws and epoxy (Polhemus, Liberty System Colchester, Vermont). The electromagnetic tracking system provides positional information with 6 degrees of freedom with an update rate of 240 Hz per a sensor and with .76 mm positional and .15 degrees of RMS accuracy.

The specimens were pre-stressed in each direction 10x to pre-stress soft tissues. The ankle was taken to the end feel in internal/external rotation and plantar/dorsiflexion by an examiner for the pre-stress protocol. All syndesmotic structures were directly inspected visually to ensure no prior trauma, surgery, or other confounding factor. Additionally, radiographs were taken to screen specimens for prior trauma. Nine of the eleven specimens made it through the complete study protocol and were included in the study. The two specimens that failed to complete the study had early fibula fracture through the tracking sensor screw holes on the fibula.

### In vitro simulation-ankle rig

**Fig. 1:**
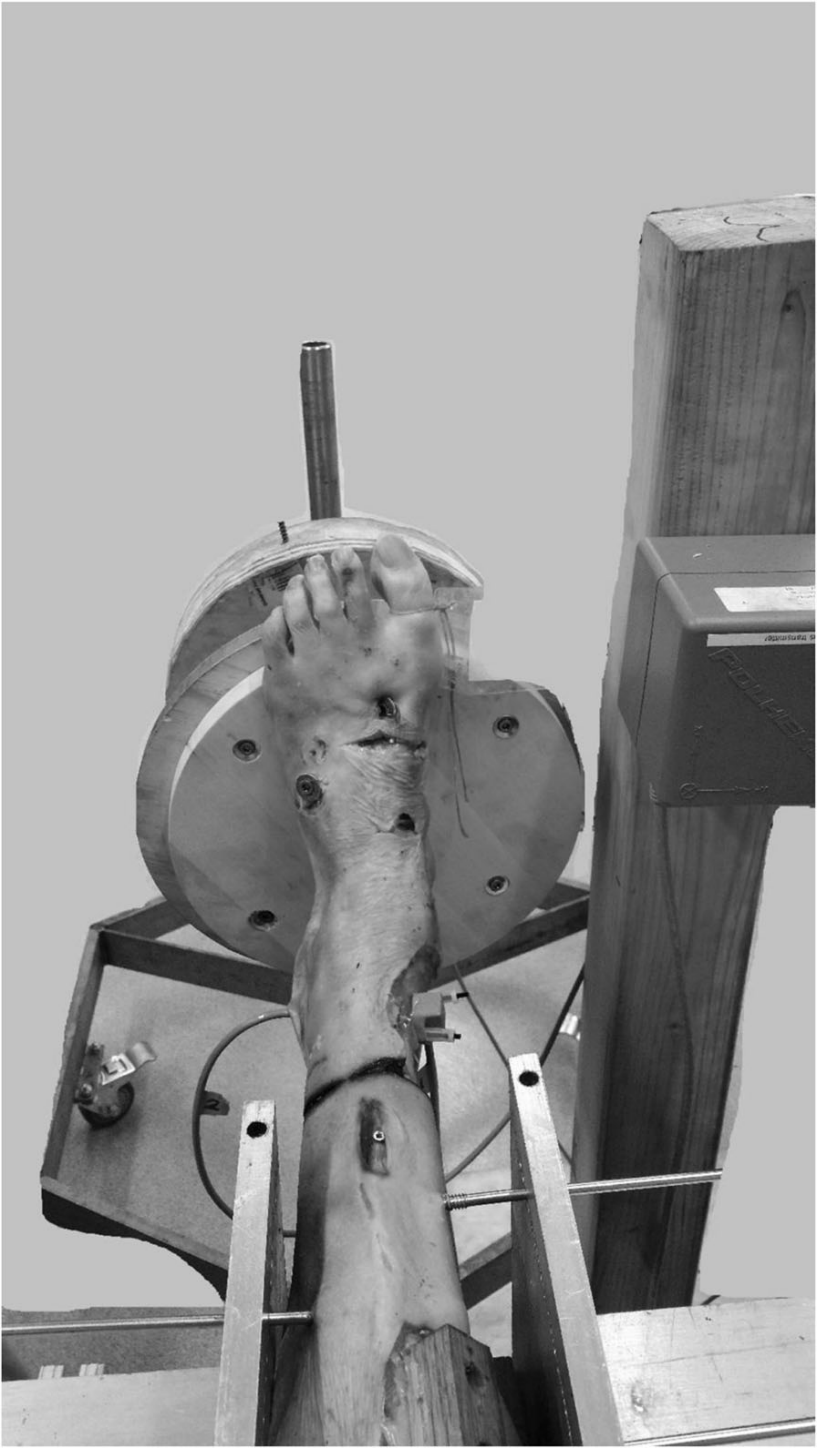
In vitro simulation ankle rig used for dynamic ultrasonography evaluation

An ankle testing rig designed to fix the tibia and allow free fibular movement was used to perform a controlled external rotational stress test that holds the foot fixed in 5 degrees of freedom while allowing rotation in the transverse plane (Fig. 1). Torque was recorded via a sensor embedded in the foot-mounting block, and ankle position was recorded using an electromagnetic tracking system. The tracking system was used solely for foot positioning acting as an electronic goniometer. No muscle loads or axial forces were applied.

**Table 1:** Lauge-Hansen SER Stages, where each stage of injury is in addition to the prior

Stage 1	AITFL
Stage 2	Fibula Fracture (Fx)
Stage 3	PITFL or Posterior Malleolar Fx
Stage 4	Deltoid or Medial Malleolar Fx

### Study protocol

The ankle was held in 15 degrees of dorsiflexion for all phases of external rotational stress testing. The examiner placed the ultrasound probe directly over the AITFL of the ankle on the skin for full visualization during testing [7]. Placement of the probe was marked on the skin with a marker using the direct visualization through the incision to identify the appropriate area 1 cm proximal to the tibiotalar joint. Ultrasound gel was used in the wound and the probe was adjusted via examiner to best visualize the tibiofibula joint with the probe perpendicular to the joint line.With the ankle held in dorsiflexion, 10 Nm of torque was achieved over a 10 s period while dynamically recording ultrasound video with a single fluoroscopic image being taken at peak torque. A ruler was used to measure the width of the ligaments and this measurement was used to calculate the necessary amount of transection required to perform a controlled partial ligament injury with a scalpel. A live digital torque readout was synchronized with the ultrasound video to allow controlled application of torque by the examiner and allowed the correct pairing of torque, time, and ultrasound image pairing via data analyses. Prior to this dynamic phase, a fluoroscopic anterior-posterior (AP) view of the ankle was taken while in neutral position. Each phase was repeated three times and averaged. This process was repeated for each of the phases listed in Table 1. These phases follow the Lauge-Hansen SER injury pattern with incremental phases of 75% ligament injury between each stage.

Digital imaging measurement software (ImageJ NIH, Bethesda MD) was calibrated to images using a ball bearing for fluoroscopic images and the on-image ruler for ultrasound images and used to measure tibiofibular clear space [7]. The tibiofibular clear space was measured by three independent observers and averaged for each phase. At each phase there were three repeated clear space length data points collected each of which were measured by the three independent obervers. The images were randomized and observers were blinded to which ankle and what level of injury were being measured. The examiner was a orthopaedic surgical resident and expert in ankle kinematics with extensive ultrasound experience. Observers were medical students who underwent training with digital measuring software to measure from point to point as described in prior literature [8]. Medical students did not participate in probe technique or clinical aspects of the study, they were solely used for digital measurement of length on recorded ultrasound images designated by the examiner.

### Intraobserver error and repeatability

Since three observers collected tibiofibular clear space measurements, an interobserver analysis was first performed using the Bland-Altman method. This statistical method compares the measurements of Observer 1 to Observer 2, Observer 1 to Observer 3, and Observer 2 to Observer 3, in a pairwise fashion. The 95% confidence interval for the mean difference between observers was used to assess interobserver error, with a null hypothesis of the mean difference between observers being 0.0 mm. All observer data was included in the analysis of experimental data as the differences amongst the oberservers was found to be similar as shown in Table 2 and in the Additional file 1.

**Table 2:** Summary of Bland-Altman analyses of average difference in tibiofibular clear space measurements. AITFL – anterior inferior tibiofibular ligament, PITFL – posterior inferior tibiofibular ligament

Injury Phase	Observer 1 vs. Observer 2	Observer 1 vs. Observer 3	Observer 2 vs. Observer 3
Normal	3.64	2.32	-1.32
75 AITFL	2.47	1.90	-0.40
100 AITFL	3.06	2.88	-0.22
Fibula Fracture	2.96	2.79	-0.17
75 PITFL	1.83	2.43	0.58
100 PITFL	1.18	1.77	0.59
Mean Average (Std. Dev.)	2.52 (0.89)	2.35 (0.45)	-0.16 (0.71)

### Data analysis

Descriptive statistics were used to establish means and a histogram and Tukeys were used to screen for potential outliers. The tibiofibular clear space of the specimens was recorded and analyzed using repeated measures one-way analysis of variance (ANOVA) on data with a Log_10_ transformation to help reduce the effect of outliers. Stress examination at each phase of injury was repeated three times and all clear space measurement were made by three different examiners and averaged to reduce error. A one-way repeated measures ANOVA with Bonferroni was used. Significance set as *P* < .05 for all data analysis.

## Results

**Fig. 2:**
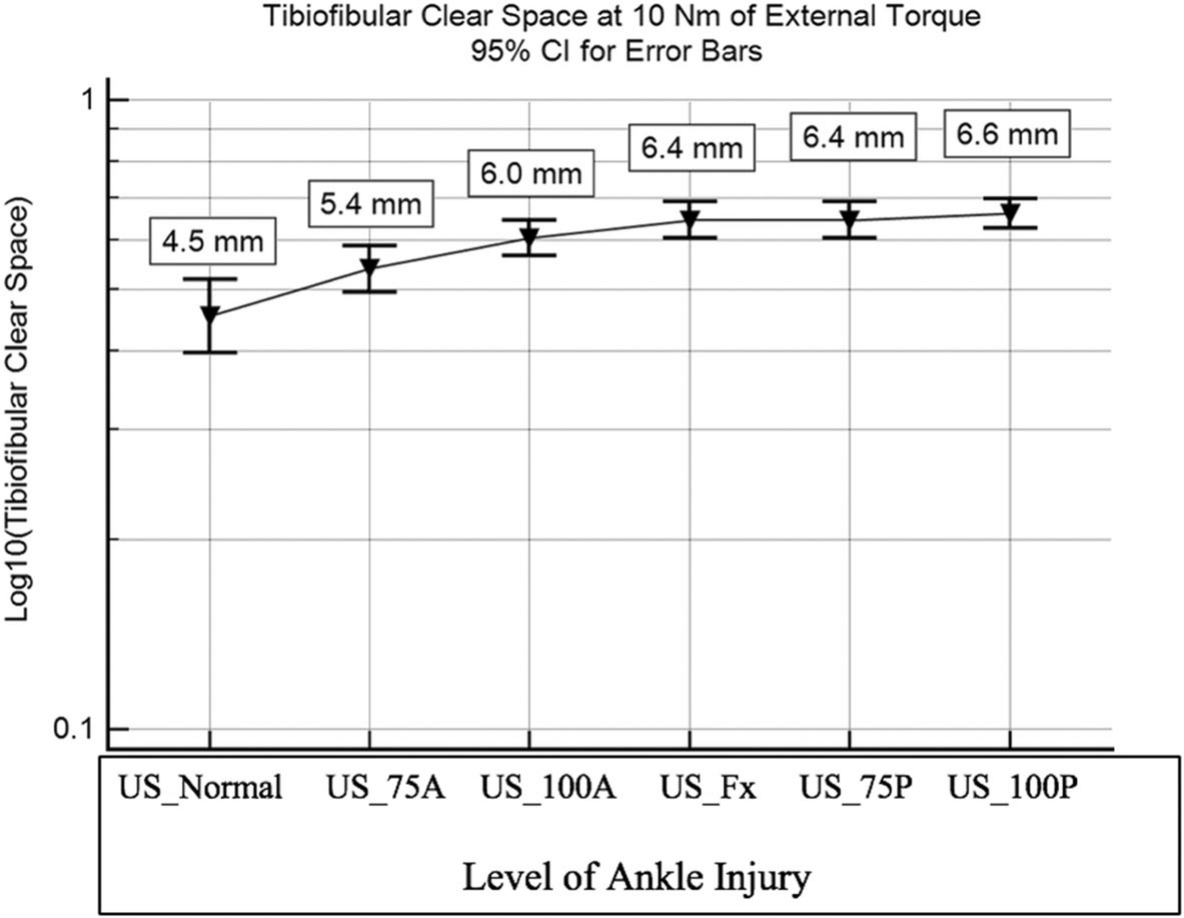
Tibiofibular clear space measurements at 10 Nm of external rotational torque at each injury level experienced in the experiment. Uninjured injury level (US_Normal); 75% of AITFL cut injury level (US_75A); 100% AITFL cut injury level (US_100A); Fibula fracture 8 cm proximal to plafond injury level (US_Fx); 75% PITFL cut injury level (US_75P); 100% PITFL cut injury level (US_100P)

The normal ankle state was found to be statistically different from the 100% AITFL injury state (*P* < .005), fibula fracture state(*P* < .005), 75% PITFL injury state (*P* < .005), and 100% PITFL injury state (*P* < .005). There was no significant difference between the normal ankle state and the 75% AITFL injury state (*P* = .107). The 75% AITFL injury state was found to be statistically different from the fibula fracture state (*P* = .046), 75% PITFL injury state (*P* = .007), and the 100% PITFL injury state (*P* < .005). There were no significant differences between the 100% AITFL injury state, fibula fracture state, 75% PITFL injury state, and 100% PITFL injury state as seen in Fig. 2. The stage 3–4 injuries seemed to plateau in clear space widening in the study as the ankle became highly unstable without muscle forces and it proved to be increasingly difficult to clearly identify a tibiofibular clear space due to the position of the bone.

## Discussion

**Fig. 3:**
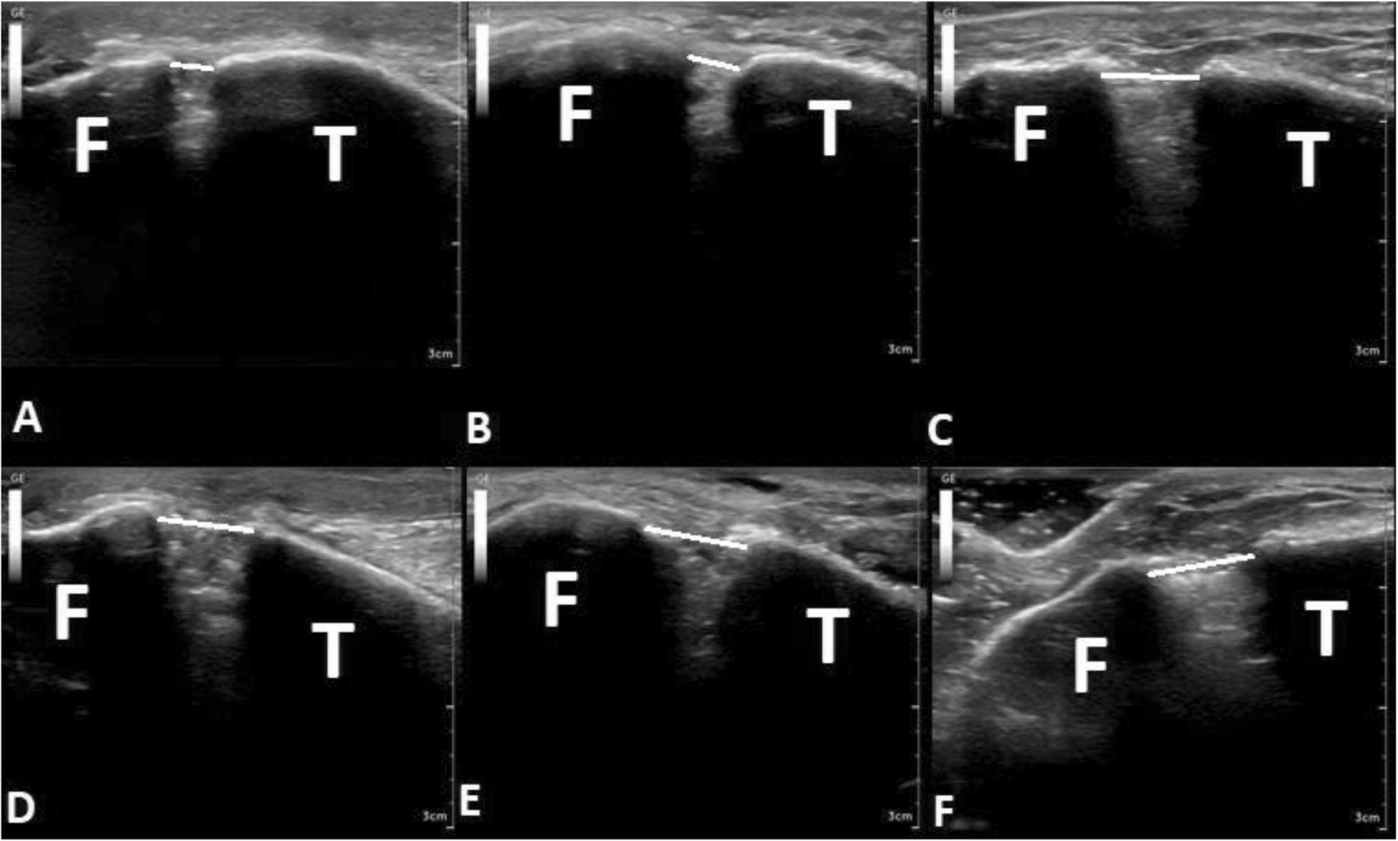
Ultrasound imaging of the tibiofibular clear space at 10 Nm of external rotational torque. White line indicates tibiofibular clear space. **a** Intact; (**b**) 75 AITFL; (**c**) 100 AITFL; (**d**) Fibula Fracture; (**e**) 75 PITFL; (**f**) 100 PITFL; [T] Tibia; [F] Fibula

This study presents evidence which supports the use of dynamic ultrasonography examination for early stage SER type syndesmosis injuries using a cadaveric model to examine the tibiofibular clear space. Our model tested the ability to detect complete and incomplete SER injuries at Lauge-Hansen stages 1–4. This is evidence that a partially intact AITFL is enough to prevent an overt diagnosis of a syndesmosis injury as the mean tibiofibular clear space in this study only widened to 5.4 mm from the 4.5 mm uninjured ankle Figs. 2-3. An AITFL with only 25% of its fibers intact was able to maintain ankle stability with a torque of 10 Nm which may indicate that patients with a partial AITFL injury would be able to weight bear as tolerated and only have activity restrictions with bracing or splinting for non-operative management at this partial level of injury. This concept would need further study to be proven as our model did not test the repetitive stress encountered with physiological movement. Additionally, a complete stage 1 injury with 100% of AITFL torn was identified with dynamic ultrasonography with 6 mm of mean tibiofibular clear space widening and was statistically different from the uninjured state. This reinforces the typical mean 6 mm tibiofibular clear space cut off for diagnosing a syndesmosis injury [8]. The addition of a fibula fracture with a stage 2 SER injury increased mean tibiofibular clear space from the stage 1 injury although less than expected (6.0 to 6.4 mm). The lack of major mean clear space widening between stage 1 and 2 may be due to AITFL being 100% cut as it is the major external rotational restraint to fibula motion and cutting the fibula had little effect during an external rotational force [9]. The stage 3–4 injuries seemed to plateau in clear space widening in the study as the ankle became highly unstable without muscle forces and it proved to be increasingly difficult to clearly identify a tibiofibular clear space due to the position of the bone. These data indicates dynamic ultrasonography evaluation can be relied upon for detection of syndesmosis disruptions of complete stage 1 injuries and above which may prevent the need for further imaging. This study was the first to show the ability to detect significant changes in tibiofibular clear space at each level of injury in a cadaveric model. Our data also indicates that a partial AITFL tear may go undiagnosed when relying upon mean clear space measurements alone with ultrasonography, as the injury did not cause a clear space widening above the 6 mm cut off typically used.

Clinically, plain film non-stress and external rotation stress radiographs, are most commonly used for assessment of syndesmosis integrity as they are low-cost and widely available.. Dynamic ultrasound evaluation of the ankle syndesmosis is also widely available and is inexpensive, but underutilized. Plain film radiography has accuracies of 48 and 64% in AP and mortise views, respectively, which reflects the limitations of fixed, single plane imaging to identify complex geometric anatomical changes [10]. Arthroscopy has the highest sensitivity and specificity of close to 100%, but is invasive and costly [10]. Evaluation through MRI, CT, and plain radiographs provide further evaluation without the invasiveness of arthroscopy (in descending order of sensitivity/specificity) [4, 5, 11, 12]. Stress examination using ultrasonography or fluoroscopy increases the value of the base test by measuring tibiofibular and medial clear spaces while syndesmotic ligaments are under tension [4, 7, 8, 12]. Dynamic stress ultrasonography of the ankle syndesmosis has most recently been reported to have a sensitivity and specificity of 100%, but the study had a small sample size [7]. An older study using less advanced ultrasound technology and comparing results to MRI reported a sensitivity of 66% and a specificity of 91% for AITFL injuries [13]. At the present time, ultrasonography is underutilized, but is inexpensive and time efficient [8]. The underutilization of dynamic ultrasonography may be due to familiarity of many surgeons with the use of fluoroscopy, with an absence of familiarity with dynamic ultrasonography, and a lack of studies directly comparing dynamic ultrasonography with stress fluoroscopy. Our study may provide some confidence to physicians for the use of dynamic ultrasonography and also highlights the need for a study directly comparing stress fluoroscopy and dynamic ultrasonography to be performed to bridge the next gap in knowledge for accurate syndesmosis injury diagnosis.

There were several limitations to this study. The fresh frozen cadaveric material had a mean age that is older than the prototypical demographic for syndesmosis injury, with a range of 41–81. Additionally, the specimens did not have muscles forces acting upon them or undergo any physiologic movement. These factors may have affected the ability to detect changes in clear space measurement, but are common limitations amongst in vitro simulations of the syndesmosis [2, 9, 14–17]. There were three independent measurements made off of one set of imaging performed by one examiner to help lessen the effect of examiner bias, which is known to affect tibiofibular clear space measurements with ultrasonography. Furthermore, findings of this cadaveric study need to be backed up by controlled trial studies. The generalizability of this study is limited, as only the supination-external rotation ankle Lauge-Hansen injury pattern was examined and there are many other injury patterns that are possible and may affect the diagnostic success of dynamic stress ultrasonography examination.

## Conclusion

Dynamic external rotational stress evaluation using ultrasonography was able to detect stage 1–4 Lauge-Hansen SER injuries with statistical significance and corroborates criteria for diagnosing a syndesmosis injury at ≥6.0 mm of tibiofibular clear space widening. These findings should build confidence with physicians and technicians in using ultrasonography on a more widespread basis, as our findings are novel. Prior clinical and biomechanical studies have only shown the sensitivity and specificity of ultrasonography diagnosis of syndesmosis injuries without much regard to severity of injury. These findings should serve as a basis for expanding the role of dynamic ultrasonography evaluation of the syndesmosis for providers and technicians.

## Supplementary information

**Additional file 1.** Interobserver Analysis; Bland-Altman interobserver analysis was completed for this project. It includes a description as to how this was done, as well as a table showing a summary of the analyses. (DOCX 14 kb)

**Additional file 2.** Letter from Vice President of the State Anatomical Board of Texas regarding declaration of Ethics Approval and Consent to Participate. (DOCX 1347 kb)

## Abbreviations

AITFL: Anterior inferior tibiofibular ligament
ANOVA: Analysis of variance
AP: Anterior-posterior
CT: Computed tomography
Fx: Fracture
IOL: Interosseous ligament
ITL: Inferior transverse ligament
MRI: Magnetic resonance imaging
PITFL: Posterior inferior tibiofibular ligament
SER: Supination-external rotation
