# Supplementary material for: Diagnostic capability of dynamic ultrasound evaluation of supination-external rotation ankle injuries: a cadaveric study
Source: BMC Musculoskelet Disord. 2019 Oct 30;20:502. doi: 10.1186/s12891-019-2899-z (PMC6822462; doi:10.1186/s12891-019-2899-z)
Supplement: Supplementary file 1 — Additional file 1. Interobserver Analysis; Bland-Altman interobserver analysis was completed for this project. It includes a description as to how this was done, as well as a table showing a summary of the analyses. (DOCX 14 kb) [file 12891_2019_2899_MOESM1_ESM.docx]

Since three observers collected tibiofibular clear space measurements, an interobserver analysis was first performed using the Bland-Altman method. This statistical method compares the measurements of Observer 1 to Observer 2, Observer 1 to Observer 3, and Observer 2 to Observer 3, in a pairwise fashion. The 95% confidence interval for the mean difference between observers was used to assess interobserver error, with a null hypothesis of the mean difference between observers being 0.0 mm.

A summary of interobserver analyses using the Bland-Altman method for the ultrasound (US) tibiofibular clear space measurements amongst observers is provided in Table 2 The results of the pairwise comparisons indicated the tibiofibular clear space measurements of Observer 2 and Observer 3 were exceedingly similar, with an average difference of only 0.16 mm between the two observers. Conversely, the tibiofibular clear space measurements of Observer 1 were found to be consistently higher than both Observer 2 (2.5 mm) and Observer 3 (2.4 mm). Despite this tendency for Observer 1 to overestimate tibiofibular clear space dimensions compared to the other two observers, the measurements of Observer 1 were found to be internally consistent (i.e., Observer 1 overestimated Observer 2 and 3 by similar amounts at each experimental phase). Thus, the measurements of Observer 1 were still included in the calculation of the overall mean value, with the understanding that all mean measurements were similarly influenced by Observer 1’s values. Accordingly, all mean values included in the subsequent two-way repeated measures ANOVA.

**Table 2** Summary of Bland-Altman analyses of average difference in tibiofibular clear space measurements. AITFL – anterior inferior tibiofibular ligament, PITFL – posterior inferior tibiofibular ligament.

| **Injury Phase** | **Observer 1 vs. Observer 2** | **Observer 1 vs. Observer 3** | **Observer 2 vs. Observer 3** |
| --- | --- | --- | --- |
| Normal | 3.64 | 2.32 | -1.32 |
| 75 AITFL | 2.47 | 1.90 | -0.40 |
| 100 AITFL | 3.06 | 2.88 | -0.22 |
| Fibula Fracture | 2.96 | 2.79 | -0.17 |
| 75 PITFL | 1.83 | 2.43 | 0.58 |
| 100 PITFL | 1.18 | 1.77 | 0.59 |
| **Mean Average**  **(Std. Dev.)** | 2.52 (0.89) | 2.35 (0.45) | -0.16 (0.71) |
