# Supplementary material for: Diagnostic capability of dynamic ultrasound evaluation of supination-external rotation ankle injuries: a cadaveric study
Source: BMC Musculoskelet Disord. 2019 Oct 30;20:502. doi: 10.1186/s12891-019-2899-z (PMC6822462; doi:10.1186/s12891-019-2899-z)
Supplement: Supplementary file 2 — Additional file 2. Letter from Vice President of the State Anatomical Board of Texas regarding declaration of Ethics Approval and Consent to Participate. (DOCX 1347 kb) [file 12891_2019_2899_MOESM2_ESM.docx]

October 14, 2019

Dear Dr. Amanpreet Athwal,

As a representative of the State Anatomical Board of Texas, I would like to address the questions you have regarding the manuscript of Dr. Cara Fisher.

In the Texas Administrative Code, Title 25, Part 4, Chapter 479, Rule 479.5(a)(1), it states the “dissection of human cadaveric material in health science, and related, education and research, and activities found by the board to be related to dissection are a special privilege and are legally authorized for members and students of the health, and related, professions for the purpose of the advancement of knowledge in these fields”. Use of cadaveric material in the state of Texas for these purposes, which includes research at the Health Science Center, does not require approval from an ethical committee, nor from an IRB.

We reached out to the North Texas Regional IRB, the governing agency of all human research projects in our area, and were given confirmation from Tania C. Ghani, MS, CIP, Assistant Director, that use of cadaver material does not require prior IRB approval. Their requirement for IRB approval is for studies using “live” individuals, not the deceased.

We have been publishing research projects in journasl for a decade or more, and these studies have always adhered to the ethical policies set forth by the State Anatomical Board of Texas. The same Texas Administrative Code referenced above states “Authorized dissection shall take place under supervision of trained and qualified persons, and only in specialized locations that have been approved by the board and which meet the standards set forth in 479.3 of this title. Bodies, or parts, of a body, shall not be removed from the specified locations without permission of the board or of an authorized representative of the board”.

Each facility is required to undergo a full inspection by the Anatomical Board of the State of Texas every five years, or if major renovations to their facility have been completed. We have passed all of our state inspections for the past 35+ years and were recently inspected in July of 2018 after a $2.6 million renovation of our human anatomy laboratory. We have seven faculty here who are highly qualified anatomists, and we have a staff of six embalming staff members who are licensed with the Texas Funeral Home Commission as part of our Willed Body Program. These licensed staff are required by the State Anatomical Board of Texas to properly handle and dispose of cadaveric material upon completion of research projects and education studies.

Sincerely,

Rustin E. Reeves, PhD

Vice Chairman, State Anatomical Board of Texas

Director, Center for Anatomical Science

UNT Health Science Center

3500 Camp Bowie Blvd.

Fort Worth, TX 76107
